# Expression of Genes Located on the Incompatibility Group FIB Plasmids at Transcription and Protein Levels in Iron-Modified Growth Conditions

**DOI:** 10.3389/fmicb.2021.729275

**Published:** 2021-11-05

**Authors:** Carter N. Abbott, Monique Felix, Steven L. Foley, Bijay K. Khajanchi

**Affiliations:** Division of Microbiology, U.S. Food and Drug Administration, National Center for Toxicological Research, Jefferson, AR, United States

**Keywords:** *Salmonella enterica*, IncFIB plasmid, iron acquisition systems, IutA, LBID, LBIR

## Abstract

*Salmonella enterica* strains often harbor plasmids representing several incompatibility groups (Inc) including IncFIB, which have been previously associated with carrying antimicrobial resistance and virulence associated genes. To better understand the distribution of virulence genes on IncFIB plasmids, we analyzed 37 complete whole genome and plasmid sequences of different *S. enterica* isolates from multiple serovars. Many of the sequences analyzed carried multiple virulence-associated genes, including those associated with iron acquisition systems; thus we aimed to determine how iron-rich (IR) and various iron-depleted (ID) conditions affected the transcription of iron acquisition and virulence genes including *sitA*, *iutA*, *iucA*, and enolase at different time intervals. *sitA*, *iutA*, and enolase from *S*. *enterica* that were grown in Luria-Bertani broth (LB) ID (LBID) conditions were substantially upregulated when compared to LBIR conditions. For both *S. enterica* strains that were grown at various LBID conditions, addition of 200 μM bipyridyl in the growth medium yielded the highest transcription for all four genes, followed by the 100 μM concentration. An antibody using a peptide targeting aerobactin receptor gene *iutA* encoded by IncFIB was generated and used to examine the protein expression in the wild-type, recipient, and transconjugant strain in LB, LBID, and LBIR growth conditions using Western blot analyses. A 70 KDa protein band was detected in the wild-type and transconjugant that carried the IncFIB plasmid, while this band was not detected in the recipient strain that lacked this plasmid.

## Introduction

*Salmonella enterica* is one of the major foodborne pathogens in the United States (Scallan et al., 2011). It is estimated that *Salmonella* is responsible for 1.2 million illnesses per year, resulting in approximately 23,000 hospitalizations and 450 deaths in the United States (Scallan et al., 2011). The characteristics of different serotypes of *Salmonella* have a role in the bacteria’s pathogenicity and virulence (Jones et al., 2008). Among the over 2600 *Salmonella* serotypes that have been identified, key serotypes such as Enteritidis, Typhimurium, Newport, and Heidelberg can colonize the intestines of food-producing animals and humans (Jones et al., 2008; Foley et al., 2013). *S. enterica* serovar Typhimurium is generally used as a model organism for pathogenicity studies (Ohl and Miller, 2001). *S.* Typhimurium has a multitude of genes that are responsible for its pathogenicity and virulence (Galan, 1996). Notably, *Salmonella* pathogenicity islands (SPIs) contain genes that encode for virulence factors that are necessary for infection of host cells (Gaviria-Cantin et al., 2017). Addition to the chromosomally-encoded virulence factors, many strains of *Salmonella* possess virulence associated plasmids that encode several virulence factors (Khajanchi et al., 2017). These plasmid-associated virulence factors include iron acquisition systems that are frequently located on the incompatibility group (Inc) FIB plasmids (Khajanchi et al., 2017).

Iron is an essential growth factor that is necessary for regulating virulence-associated genes (Litwin and Calderwood, 1993; Schaible and Kaufmann, 2004). Because of this feature, the iron-limited conditions that are present in the host cells must be overcome for *Salmonella* to successfully infect the host (Porcheron and Dozois, 2015). Iron acquisition for *Salmonella* is regulated by the global ferric uptake regulator (Fur) (Mey et al., 2005; Porcheron and Dozois, 2015). Fur senses the iron availability in the environment and regulates the expression of iron acquisition genes and/or virulence genes, directly or indirectly (Mey et al., 2005; Porcheron and Dozois, 2015). In most cases, Fur represses iron acquisition genes under iron-rich conditions (Mey et al., 2005). The iron acquisition systems for *Salmonella* can be located on both the chromosome and plasmids (Litwin and Calderwood, 1993; Di Lorenzo and Stork, 2014). These iron acquisition systems allow for *Salmonella* to establish infection by chelating iron from the host (Page, 2019). It has been demonstrated that the chromosome-encoded Sit iron transport system contributes to the virulence in *S.* Typhimurium with the *sitABCD* operon being induced in iron-depleted conditions and the Sit system being expressed at higher levels after the invasion of intestinal cells (Janakiraman and Slauch, 2000).

The IncFIB plasmids have been shown to encode for an analogous Sit iron acquisition system, the aerobactin iron acquisition system (*iucABCD-iutA*) as well as *iroBCDEN* iron acquisition system (Han et al., 2013; Khajanchi et al., 2017). The regulation mechanism for the plasmid-encoded Sit and aerobactin iron acquisition systems are not well understood. In a recent study, we demonstrated that the IncFIB plasmid likely increases the virulence by improving the persistence of *Salmonella* in intestinal cells (Khajanchi et al., 2017). In an additional study, we showed that iron concentration in growth media impacted the global gene expression on several iron acquisition and virulence genes (Khajanchi et al., 2019). In this study, we aimed to further our understanding of how iron-rich and various iron-depleted growth media conditions affect the transcriptional and translational levels of selected iron-acquisition and virulence genes located on the IncFIB plasmids.

## Materials and Methods

### Bacterial Strains and Growth Media

*Salmonella enterica* wild type SE163A, recipient SE819, and a transconjugant (SE819:IncFIB) were used in this study. SE163A and SE819 were isolated from turkeys and previously sequenced in our laboratory (Khajanchi et al., 2016). SE163A is a *S.* Typhimurium isolate and SE819 is a *S.* Heidelberg isolate. Additionally, two *Salmonella* Typhimurium isolates from food SE426 and SEN032 and two *S.* Schwarzengrund isolates MDH29 and WLSH-7 from human patients were included for gene expression studies. Isolate SE426 originated from a contaminated turkey and SEN032 from chicken. MDH29 is isolated from urine while WLSH-7 is from gallbladder. In our earlier efforts to study IncFIB plasmids in *Salmonella*, screening of sequenced isolates from GenBank revealed that IncFIB plasmids were relatively commonly carried by *S.* Schwarzengrund isolates and the serotype is associated with causing human illnesses, hence *S.* Schwarzengrund was included in the study. The transconjugant was developed as mentioned in our previous study (Khajanchi et al., 2017). The iron-rich (IR) and iron-depleted (ID) growth media were prepared as mentioned in previous studies with ferric chloride (100 μM) and 2′,2′-bipyridyl (200 μM) (Sigma-Aldrich, St. Louis, MO) being added to LB broth, respectively (Bjarnason et al., 2003; Zaini et al., 2008; Khajanchi et al., 2019). Additionally, various LBID conditions were created by supplementing different concentrations of bipyridyl including 5, 25, 50, and 100 μM.

### *In silico* Analyses of IncFIB Plasmid Sequences of *Salmonella enterica*

**FIGURE 1:**
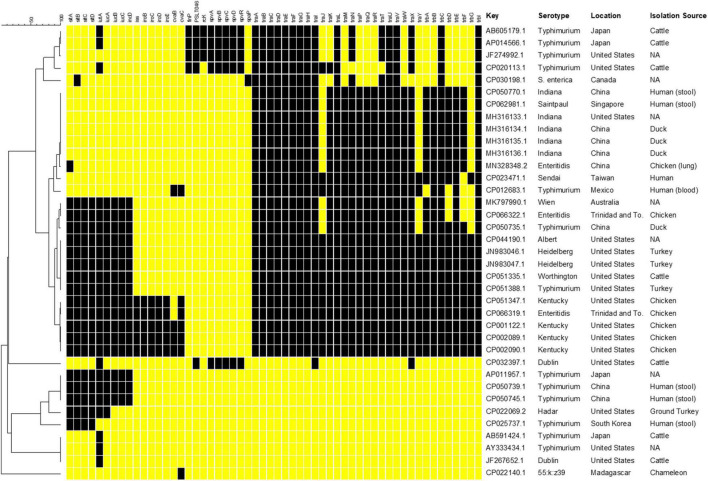
Results of *in silico* genetic analyses of sequences containing the IncFIB replicon sequences. Results are shown for the following genes: plasmid-associated *sitABCD, iutA, iucABCD, iss, iroBCDE, cvaBC, finP, rcK, spvABCDR*, and plasmid transfer associated genes. The resultant presence (black boxes) and absence (yellow boxes) data were entered into BioNumerics and the resultant phylogenetic analyses using UPGMA analysis are shown. The column with the header “Key” includes the GenBank accession number. Information on the serotype, country of origin (Location) and host species (Isolation Source) where the *Salmonella* were collected was extracted from the GenBank metadata when available. Missing information is indicated by “NA.”

The selection of IncFIB positive *S. enterica* isolates was done through an initial Microbial Nucleotide BLAST search with the IncFIB reference sequence described by Carattoli et al. (2005) against *S. enterica* (taxon ID: 28901) complete genome and complete plasmid sequences in the NCBI Complete Prokaryote Genome and Representative Plasmids Databases. Default search setting for “highly similar sequences (megablast)” were used for the BLAST searches. The resultant 37 strains that contained whole genome sequences and either an IncFIB plasmid or the IncFIB replicon integrated into the chromosome (Figure 1) were then searched for the presence of the following genes: plasmid-associated *sitABCD, iutA, iucABCD, iss, iroBCDE, cvaBC, finP, rcK, spvABCDR*, and plasmid transfer-associated genes using BLAST searching and the NCTR Virulence and Transfer Gene Databases (Aljahdali et al., 2020). The resultant presence/absence data were entered into BioNumerics (version 7.6, Applied Maths, Kortrijk, Belgium) and phylogenetic analyses were conducted using Dice correlation coefficients based on the presence of the different virulence genes to generate a dendrogram using unweighted pair group means with averages (UPGMA) clustering using the BioNumerics default settings.

### RNA Isolation

Bacterial strains were sub-cultured on sheep’s blood agar plates (Remel, Lenexa, KS). Wild type (SE163A) and the transconjugant (TC) (SE819:IncFIB) were inoculated by shaking in LB broth overnight at 37°C. The following day, 5 mL overnight cultures of each strain were pelleted by centrifugation at 6,000 RPM. Then, the bacterial pellets were subsequently resuspended using LBIR and LBID broth and were incubated at 37°C with shaking at 180 RPM for 2, 4, and 18 h. Both strains were also incubated in LBID conditions by supplementing different concentrations of bipyridyl including 5, 25, 50, 100, and 200 μM for 4 h. Two biological replicates were used for each strain at every time point for both the iron-rich and the iron-depleted growth media conditions. After each time point, the corresponding cultures were centrifuged for at 6,000 RPM at 4°C for 6 min, decanted, and stored at −20°C with RNA protector (Qiagen, Redwood city, CA) overnight. The next day, RNA was isolated from every culture with the Ribopure Bacterial RNA Isolate Kit (Ambion, Invitrogen, Carlsbad, CA). Subsequently, the genomic DNA was removed by treatment with DNase I (Ambion, Invitrogen) (Khajanchi et al., 2019). RNA samples purity was measured using a NanoDrop (Invitrogen, Carlsbad, CA) and the concentration was measured by a Qubit 4 fluorometer (Invitrogen) using Qubit RNA BR Assay kit (Invitrogen).

### Quantitative Reverse Transcription-PCR

cDNA was synthesized using 75–250 ng of RNA using the iScript cDNA synthesis kit (Bio-Rad, Hercules, CA) (Khajanchi et al., 2017). Quantitative reverse transcription PCR (qRT-PCR) was performed on the synthesized cDNA with SYBR green assays using the CFX Touch real-time PCR detection system (Bio-Rad). qRT-PCR was conducted to evaluate gene expression in four genes: *sitA*, *iutA*, *iucA*, and enolase. The gene Id for enolase is AY603_24505 (Khajanchi et al., 2019). We used “enolase” for simplicity and clarity all thorough manuscript.

The *gmk* and *adk* genes of *S. enterica* were used as reference genes to normalize gene expression (Khajanchi et al., 2019). The primers were designed using the PrimeQuest tool (Integrated DNA Technologies, Coralville, IA) (Khajanchi et al., 2017, 2019) and were specifically designed to only detect the *sitA*, *iutA*, and *iucA* genes located on the IncFIB plasmid, rather than the chromosome. Differential gene expression was determined using CFX manager software (Bio-Rad).

### Antibody Development and Western Blot Analysis

An IutA antibody was developed at Thermo Fisher Scientific Laboratory (Thermo Fisher Scientific, Waltham, MA) using poly-clonal rabbit custom antibody protocol. Briefly, 18 amino acid long peptide at C-terminal of IutA was selected, synthesized, conjugated with Keyhole limpet hemocyanin (KLH), a copper-containing non-heme protein (Supplementary Figure 1). Subsequently, peptide-protein conjugate was injected subcutaneously into two pathogen-free New Zealand white rabbits, then boosted at Days 14, 28, 42, 56, and a final boost at Day 90 and sacrificed on Day 120. Serum was collected at Days 56, 72, and 120 (terminal bleed) and antibody titer was measured using ELISA. Two batches of sera (Days 72 and 120) were subjected to affinity purification. All the above steps were performed at the Thermo Fisher Scientific Laboratory using standard protocols.

Western blot was performed using affinity purified poly clonal anti sera collected from two rabbits in our laboratory (Bekele et al., 2016). Wild type, recipient, and transconjugant *Salmonella enterica* were grown in LB, LBID, and LBIR as described in section “Bacterial Strains and Growth Media.” Lysate was prepared from cell pellet collected by centrifugation of different bacterial cultures after 4 h of growth. Cell pellets were suspended using SDS sample buffer (Laemmli 2× or 4×), boiled, and run using 4–20% strain free TGX mini gel (Bio-Rad) at constant 200 mV for 25–30 min.

Proteins were transferred on to PVDF membrane by a Trans-Blot Turbo system using Bio-Rad Turbo Transfer kit for mini gel. After transfer, the membrane was briefly washed using Tris- buffered saline (TBS) and then the membrane was blocked for 1–2 h using 1% casein blocker. The primary antibody was diluted 1:200 using 0.1% casein blocker, and the membrane was incubated overnight at 4°C. The next day, the membrane was washed 5 times for 5–10 min each time using wash buffer TBS with 0.05% Tween 20 (TBST) and incubated with diluted secondary antibody (goat anti-rabbit-HRP) (1:5,000) and Precision Protein^TM^ StrepTactin-HRP conjugate (1:500) for 1 h. The membrane was washed 5 times for 5–10 min each time using the TBST and a final wash using TBS to remove tween from the membrane. For detection, the membrane was incubated with Clarity Max Western ECL substrate for 5 min and the blot was activated for chemiluminescence and photographed using ChemiDoc MP (Bio-Rad). For further confirmation of three protein bands that were detected by the chemiluminescence method, we performed another sensitive detection method using StarBright B520. StarBright B520 goat anti-Rabbit secondary antibody, after diluting with TBS 0.1% casein blocker (1:2,500), was used in an independent experiment. Pre-immunized serum was used as a negative control. All the antisera collected at different intervals were investigated; however, only representative blots with affinity purified sera are presented in the manuscript.

## Results and Discussion

### *In silico* Analyses of Virulence Genes Located on the IncFIB Plasmids

The number of studies that have examined the virulence-associated characteristics related to IncFIB plasmids in *Salmonella enterica* is quite limited, with much of the focus on the Spv locus that contributes to virulence in a subset of serotypes (Gulig et al., 1993; Rotger and Casadesus, 1999). Other studies have focused on contribution of related plasmids to virulence in avian pathogenic *E. coli.* Therefore, a goal of this study was to build on earlier efforts of the research team (Han et al., 2013; Khajanchi et al., 2017, 2019) and conduct further analyses of the virulence-associated genes in *Salmonella* strains. To initiate these analyses and gain a better understanding of the genetics of IncFIB plasmids present in *S. enterica*, complete plasmid and WGS sequences from *Salmonella* that were found to carry the IncFIB replicons, were analyzed for the presence of genes that have been associated with IncFIB and related plasmids (Figure 1). Complete genome and plasmid sequences were chosen for the analyses to avoid challenges with draft sequences and the linking of specific genes on sequence contigs to specific genetic elements (e.g., plasmid or chromosomes) that could confound the analyses. Of the sequences analyzed, four of the IncFIB-associated sequences, three *S.* Typhimurium (AP011957.1, CP050739.1, and CP050745.1) and an *S.* Hadar (CP022069.2) strains appeared to be integrated into the chromosomes of strains, located within insertion sequence (IS) elements, which is indicative of horizontal gene transfer. In these instances, each *S.* Typhimurium strain carried the plasmid-associated Sit (*sitABCD*) and aerobactin (*iutA, iucABCD*) operons, while the *S.* Hadar isolate lacked *iucBCD*. It is important to note that most *Salmonella* strains carry a chromosomally located Sit operon, which is distinct from the plasmid-encoded version targeted in this study (Han et al., 2013; Khajanchi et al., 2017). None of these strains had any of the plasmid transfer-associated genes.

Five of the plasmids were “traditional” *Salmonella* virulence plasmids, with the Spv operon (Gulig et al., 1993). These include four plasmids isolated from *S.* Typhimurium and one from *S.* Dublin, which are serotypes that are often associated with carrying the Spv plasmids (Rotger and Casadesus, 1999). Interestingly, the *S.* Dublin plasmid (CP032397.1) was quite unique, in that it is rather large (over 300 kB) and appeared to be a cointegrated plasmid with sequence from an IncFIB plasmid (Spv operon), as well as an IncA/C plasmid, as most of the detected transfer genes are associated with IncA/C plasmid genotype (data not shown) (Hsu et al., 2019). Each of the Spv-containing plasmids also contained some IncFIB-associated transfer genes, although not the number that many of the other plasmid sequences carried, which likely limits their ability to be transmitted to other serovars. When it comes to the IncFIB-associated transfer genes, 10 of the plasmids carried the full complement of transfer genes, while eight carried all but *traJY* and *trbG*. Twenty-seven (82% of plasmid sequences) carried *traA-I, traL, traUV, trbC*, and *trbI.* Of the 10 plasmids carrying all the transfer genes, all originated from the U.S. or Trinidad and Tobago and all but one with known sources originated from poultry (Figure 1). Interestingly, these 10 plasmids were isolated from six different serotypes, which may indicate the importance of the transfer genes for the dissemination of the plasmids among diverse *Salmonella* strains.

In addition to the plasmids carrying the Spv operons, 14 (42%) of the plasmids carried the Sit operon and 13 (40%) contained the full aerobactin operon. An additional seven plasmids (21%) carried just *iutA*, which is the ferric aerobactin receptor (Johnson et al., 2006). Five plasmids (15%) carried the genes for *iss*, which encodes the increased serum survival protein and *iroBCDEN* that encodes the salmochelin siderophore system (Nolan et al., 2003; Johnson et al., 2006; Khajanchi et al., 2017). Four of these plasmids were isolated from *S.* Kentucky and the fifth appeared to be from an *S.* Enteritidis isolate based on their GenBank records. These five plasmids, in addition to two others (*N* = 7, 21%), carried the colicin V gene (*cvaC*) and four (12%) carried the *cvaB* colicin gene. The colicins can function to kill susceptible bacteria near the strains that produce the toxins, thereby potentially providing a competitive advantage to the strain (Cascales et al., 2007). Interestingly, one of the plasmids (CP022140.1) was isolated from a *S. enterica* subspecies *salamae* strain. This particular plasmid carried several bacteriocin genes along with fimbrial-associated genes. While not the main focus of this paper, it is important to note that many of the plasmids examined also carried multiple antimicrobial resistance genes, in addition to the potential virulence associated genes, which can be a potential concern related to the co-selection of increased antimicrobial resistance and virulence (Aljahdali et al., 2020).

When the virulence factor data were parsed out based on serotype, the *S.* Typhimurium strains displayed more extensive diversity in their profiles relative to the other serotypes whose members were more closely clustered (Figure 1). This observation may be due to the relatively high number of *S.* Typhimurium sequences examined (13/37, 35% of all strains) providing more opportunity to observe diversity. Additionally, in some of the *S.* Typhimurium strains, the IncFIB-associated sequences appeared to be located on the chromosome of the strains and lacking plasmid-specific genes. In contrast, the plasmids originating from serovars Indiana (*N* = 5, 14%) and Kentucky (*N* = 4, 11%) tended to have identical or near identical profiles. Some of these similarities may be due to originating from related location and sources, as noted above for those isolates with the transfer genes, but likely not all. For example, four of the *S.* Indiana strains were sequenced in China (three from duck and one from chicken) and the fifth in the United States based on their GenBank entries.

In looking at the plasmid characteristics and metadata for the plasmids, the 10 isolates containing the complete set of transfer genes are interesting in that they all also carried the genes associated with the plasmid-associated Sit and aerobactin operons and five of these plasmids were also those that carried *iss* and the *iro* operon. These virulence factors are often found in avian pathogenic *Escherichia coli* (APEC) and are associated with extraintestinal disease (Nolan et al., 2003; Johnson et al., 2006; Khajanchi et al., 2017). In APEC from the U.S., these genes are also carried on IncFIB plasmids (Johnson et al., 2006, 2007). With the predominance of these APEC-like plasmids isolated from *Salmonella* coming primarily from poultry in the U.S., it is quite probable that there is interspecies spread of these plasmids in this poultry environment.

### Gene Expression of Iron Acquisition Genes in Iron-Rich and Iron-Depleted Growth Conditions With Various Growth Periods

**FIGURE 2:**
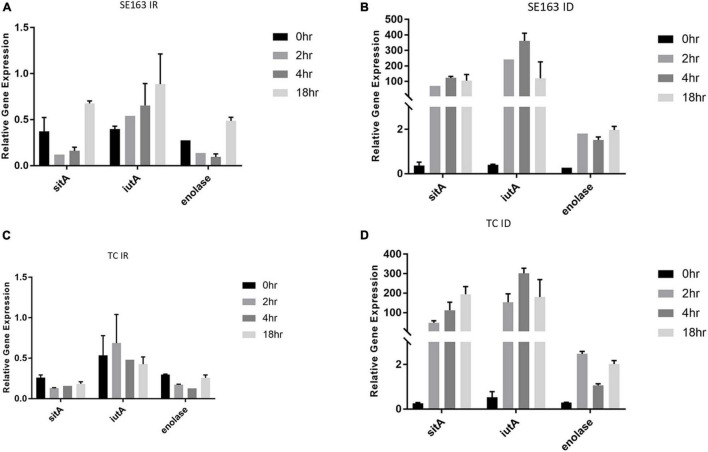
Iron acquisition and other virulence genes of *S. enterica* were upregulated in iron-depleted conditions compared to iron-rich conditions. qRT-PCR was performed using SYBR green assays on *sitA*, *iutA*, and enolase to examine gene expression levels in LBIR **(A,C)** and LBID **(B,D)** conditions. The data in the figure depicts the transcription level of either strain SE163 **(A,B)** or transconjugant (TC) **(C,D)** grown at 0, 2, 4, or 18 h. LBIR was supplemented with 100 μM ferric chloride and LBID was supplemented with 200 μM bipyridyl. The 0 h time point, where strains were grown in LB, served as the baseline expression level of these four genes. Two biological replicates and three technical replicates were used for each sample (±SD). Gene expression was normalized by *gmk* and *adk* reference genes. Student’s *t*-test was performed between 0 h vs. 2 h; 0 h vs. 4 h; 0 h vs. 18 h with data being considered significant when *p* < 0.05. Although increased expression of *sitA*, *iutA*, and enolase were noted between the groups compared (0, 2, 4, and 18 h) in iron depleted condition for both SE163 and TC strains; these data were statistically non-significant.

*S*. *enterica* was grown at various time intervals in iron-depleted and iron-rich conditions to better understands the impact that these growth conditions have on the gene expression of the plasmid-encoded *sitA*, *iutA*, and enolase over time. Increased expression of *sitA* and *iutA* were observed for both the wild-type SE163A and the transconjugant (SE819:IncFIB) in LBID conditions when compared to the LBIR conditions (Figure 2). These results show that iron-depleted conditions lead to an increase of expression of iron acquisition genes when compared to iron-rich conditions, confirming the findings from our previous study (Khajanchi et al., 2017). Furthermore, we observed differences in gene expression for these genes when examining their expression at different growth periods in iron-depleted conditions (Figures 2B,D). We observed that in iron depleted conditions, gene expression increased at 2, 4, and 18 h as compared to control at 0 h. However, this difference is statistically non-significant (Figures 2B,D). These results suggest that the length of time that *Salmonella* is present in iron-depleted conditions impacts the transcription rate of iron acquisition genes.

### Gene Expression of Iron Acquisition Genes in Various Iron-Depleted Growth Conditions

**FIGURE 3:**
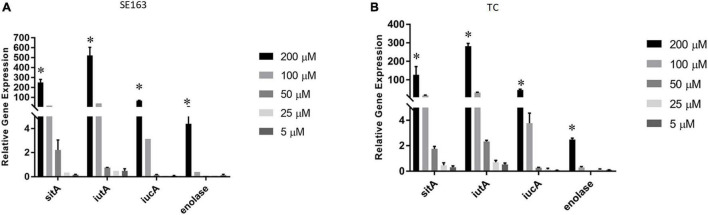
Iron acquisition and other virulence gene expressions of *S. enterica* were increased in decreasing iron concentrations. Quantitative reverse transcription-PCR (qRT-PCR) was performed using SYBR green assays on the four genes *sitA*, *iutA*, *iucA*, and enolase to determine gene expression levels in LBID. The data presented in the graph are the transcription levels of four genes from either the wild-type SE163 **(A)** or the transconjugant (TC) **(B)**. Both strains were grown for 4 h in various LBID media using different concentrations of bipyridyl. Two biological replicates and three technical replicates were used for each sample (±SD). Gene expression was normalized by *gmk* and *adk* reference genes. Student’s *t*-test was performed between 5 μM vs. 25 μM; 5 μM vs. 50 μM; 5 μM vs. 100 μM; 5 μM vs. 200 μM with data being considered significant when *p* < 0.05. Asterisk (^∗^) indicates that increased expression of 4 genes at 200 μM as compared to 5 μM of bipyridyl was statistically significant for both SE163 and TC strains.

We sought to understand how various iron-depleted growth conditions affect the expression of the plasmid-encoded *sitA*, *iucA*, *iutA*, and enolase genes. We found that all four genes were expressed higher as the concentration of bipyridyl was increased for both the wild-type and transconjugant with the strains grown in 200 μM bipyridyl having the highest expression (Figure 3). These results indicate that when these isolates are exposed to environments where iron is less readily available, they are able to increase their gene expression of their iron acquisition systems, potentially improving their ability to acquire iron.

### Impact of Iron-Depleted and Iron-Rich Conditions on the Transcription of Virulence Genes Encoded by the IncFIB Plasmids Harbor in Additional *Salmonella* Strains

**FIGURE 4:**
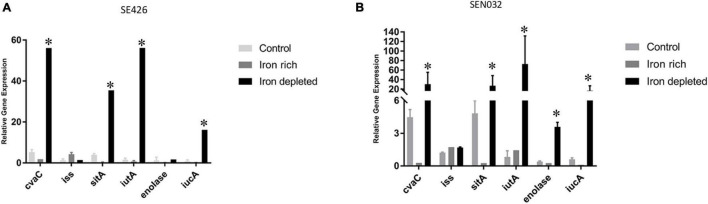
Virulence gene expressions of *S. enterica* were upregulated in iron-depleted conditions compared to iron-rich. Quantitative reverse transcription-PCR (qRT-PCR) was performed using SYBR green assays on six genes *cvaC*, *iss*, *sitA*, *iutA*, enolase, and *iucA.* The data in the figure depicts the transcription level of either strain SE426 **(A)** or strain SEN032 **(B)**. Both strains were grown for 4 h in LBID or LBIR media. The 0 h control was grown on LB and served as the baseline expression level of tested genes. Two biological replicates and three technical replicates were used for each sample (±SD). Gene expression was normalized by *gmk* and *adk* reference genes. Student’s *t*-test was performed between control vs. iron rich; control vs. iron depleted; iron depleted vs. iron rich with data being considered significant when *p* < 0.05. Asterisk (^∗^) indicates statistically significant difference between expression of genes at iron depleted as compared to iron rich condition.

In order to determine how iron-depleted and iron-rich growth conditions impact the expression of virulence genes in the *Salmonella* isolates SE426 and SEN032, we performed qRT-PCR on six genes *cvaC*, *iss*, *sitA*, *iutA*, enolase, and *iucA*. *iss* (increased serum survival) is a virulence factor commonly found in APEC; *sitABCD*, is an ABC-type transporter that transports iron and manganese where *sitA* functions as periplasmic metal ion binding protein; *iucABCD*-*iutA* is an aerobactin operon that is involved in aerobactin synthesis and transport where *iucA* functions as synthetase; *iutA* is a ferric aerobactin receptor protein and is also involved in virulence; enolase is a virulence factor which may be involved in invasiveness and tissue damage; and *cvaC* is a colicin V synthesis protein, colicin V has bactericidal activity. Our results showed that there was an increase in gene expression for genes *cvaC*, *sitA*, *iutA*, and *iucA* in both isolates grown in LBID when compared to the 0 h control grown in LB (Figures 4A,B). We also observed a significant increase in expression for *enolase* in SEN032 for iron-depleted growth when compared to control (Figure 4B). Expression of *iss* showed little or no change as compared to the 0 h control, indicating that expression of this gene is not regulated by the iron modified growth conditions. *sitA*, *iutA*, *iucA*, and *cvaC* were increasingly expressed under iron depleted condition as compared to iron rich condition for both SEN032 and SE426 isolates, and this difference was statistically significant. We also determined the gene expression of *sitA* and *iutA* from two *S.* Schwarzengrund human isolates (MDH 29 and WLSH 7) containing IncFIB plasmids in LBID and LBIR using qRT-PCR. Similar to *S.* Typhimurium, increased expression of *sitA* and *iutA* were detected in LBID as compared to LBIR (Supplementary Figures 2A,B). Overall, these data indicate that iron acquisition system genes encoded by IncFIB plasmids carried by different *Salmonella* serovars isolated from food and human sources demonstrated similar expression profiles in response to iron concentrations in the growth media.

### Determine the Expression of IutA Protein in Luria-Bertani Broth, Luria-Bertani Broth Iron-Depleted, and Luria-Bertani Broth Iron-Rich

**FIGURE 5:**
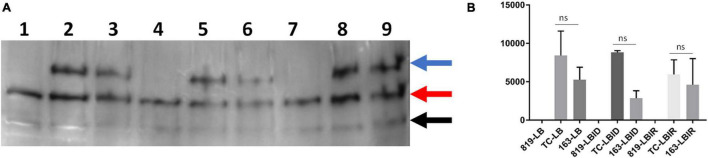
Western blot analyses of IutA encoded by IncFIB plasmid. **(A)** Protein expression was detected by chemiluminescence. Lanes 1, 4, 7: protein lysate prepared from the recipient strain-SE819; Lanes 2, 5, 8: from transconjugant (SE819:IncFIB); Lanes 3, 6, 9: from wild type-SE163. Lanes 1–3: All three strains grown in LB; lanes 4–6: were grown in LBID; Lanes 7–9: grown in LBIR. Three proteins were detected by western blot analyses as indicated by the blue arrow, a protein of ∼70 KDa; red arrow a protein of ∼50 KDa, and black arrow a protein of ∼30 KDa. This is a magnified area of an original blot that is shown in Supplementary Figure 3. **(B)** Band 1 (∼70 KDa) was quantified from three independent blots by ImageJ. Student’s *t*-test was performed between TC-LB vs. 163-LB; TC-LBID vs. 163-LBID; TC-LBIR vs. 163-LBIR with data being considered significant when *p* < 0.05. Although expression of IutA was decreased in SE163 as compared with transconjugant in all three conditions (LB, LBID, and LBIR), however; this difference was statistically non-significant.

A ∼70 KDa protein was detected (indicated by the blue arrow in Figure 5 and Supplementary Figure 4) in wild type-SE163 and transconjugant (SE819:IncFIB) *S. enterica* strains in all three growth conditions-LB, LBID, and LBIR. This band was absent in the recipient strain (SE819) that lacked IncFIB plasmid indicating that a 70 KDa protein is the IutA protein encoded by the IncFIB plasmid. Two other protein bands approximately 50 and 30 KDa (indicated by red and black arrow, respectively, in Figure 5 and Supplementary Figure 4) were present in all three strains under all three tested growth conditions. These data suggest that these two bands are likely two proteins or break down products of a protein encoded by the chromosome that has some similarity to IutA.

Expression of IutA was decreased in LB, LBID, and LBIR in wildtype-type SE163 background as compared to transconjugant background (Figure 5B) suggesting that IutA may be regulated differently in these two strains. It appears that a base level of expression of IutA was observed in all three tested conditions (LB, LBIR, and LBID). We did not detect increased expression of IutA in LBID as compared to LBIR and LB, possibly because we only tested at 4 h of iron exposure for this experiment. Increased expression may occur at an earlier or later time point.

None of the protein bands were detected when using pre-immunized (Day 0) sera in the either chemiluminescence or StarBright detection methods (Supplementary Figures 5, 6). These results indicated that bands that were detected using IutA antisera specifically reacts with IutA or similar protein(s). Our study is first to develop IutA antibody by targeting IutA protein located on the IncFIB plasmid. For this we were cautious and used two detection methods, both chemiluminescence (Figure 5) and StarBright blue B520 (Supplementary Figure 4) in order to verify the specificity of detection of each method and evaluate the consistency of the findings.

The role and functions of chromosome-mediated iron acquisition protein(s) are well studied; however, the role and functions of plasmid (e.g., IncFIB) mediated iron acquisition proteins, including IutA, remain largely unknown. In this study we developed antibody against IutA and detected the protein expression of IutA at iron-rich and iron-depleted condition. In future, we will work to further identify the biological function characterization of IutA in *Salmonella* strains. We determined that ID conditions increase the expression of iron acquisition and other virulence genes when compared to the baseline and IR growth conditions. We also demonstrated that increasing the concentration of the iron chelator (bipyridyl; 5–200 μM) increased the expression of iron acquisition genes. Additionally, an antibody of IutA was generated and protein expression was detected in wild type and transconjugant strains of *Salmonella* grown in LB, LBID, and LBIR media.

In summary, our study demonstrated that iron-depleted growth media increases the transcription of iron acquisition and virulence genes that are located on the IncFIB plasmid. In addition, the findings showed that the time of exposure in ID growth media impacts the transcription levels of iron acquisition genes. Further study is necessary to determine the specific mechanism of how ID conditions in host cells impact gene expression of iron acquisition and/or virulence genes during infection of human epithelial (Caco-2) cells with different *Salmonella* isolates.

## Data Availability Statement

The datasets presented in this study can be found in online repositories. The names of the repository/repositories and accession number(s) can be found in the article/Supplementary Material.

## Author Contributions

CA, MF, and BK performed the experiments. CA, MF, SF, and BK wrote the manuscript. SF and BK reviewed and edited the manuscript. All authors contributed to the article and approved the submitted version.

## Conflict of Interest

The authors declare that the research was conducted in the absence of any commercial or financial relationships that could be construed as a potential conflict of interest.

## Publisher’s Note

All claims expressed in this article are solely those of the authors and do not necessarily represent those of their affiliated organizations, or those of the publisher, the editors and the reviewers. Any product that may be evaluated in this article, or claim that may be made by its manufacturer, is not guaranteed or endorsed by the publisher.
