# Supplementary material for: Expression of Genes Located on the Incompatibility Group FIB Plasmids at Transcription and Protein Levels in Iron-Modified Growth Conditions
Source: Front Microbiol. 2021 Nov 5;12:729275. doi: 10.3389/fmicb.2021.729275 (PMC8602916; doi:10.3389/fmicb.2021.729275)
Supplement: Supplementary file 1 [file Table_1.DOCX]

# Figure S1

#

# Figure S2A


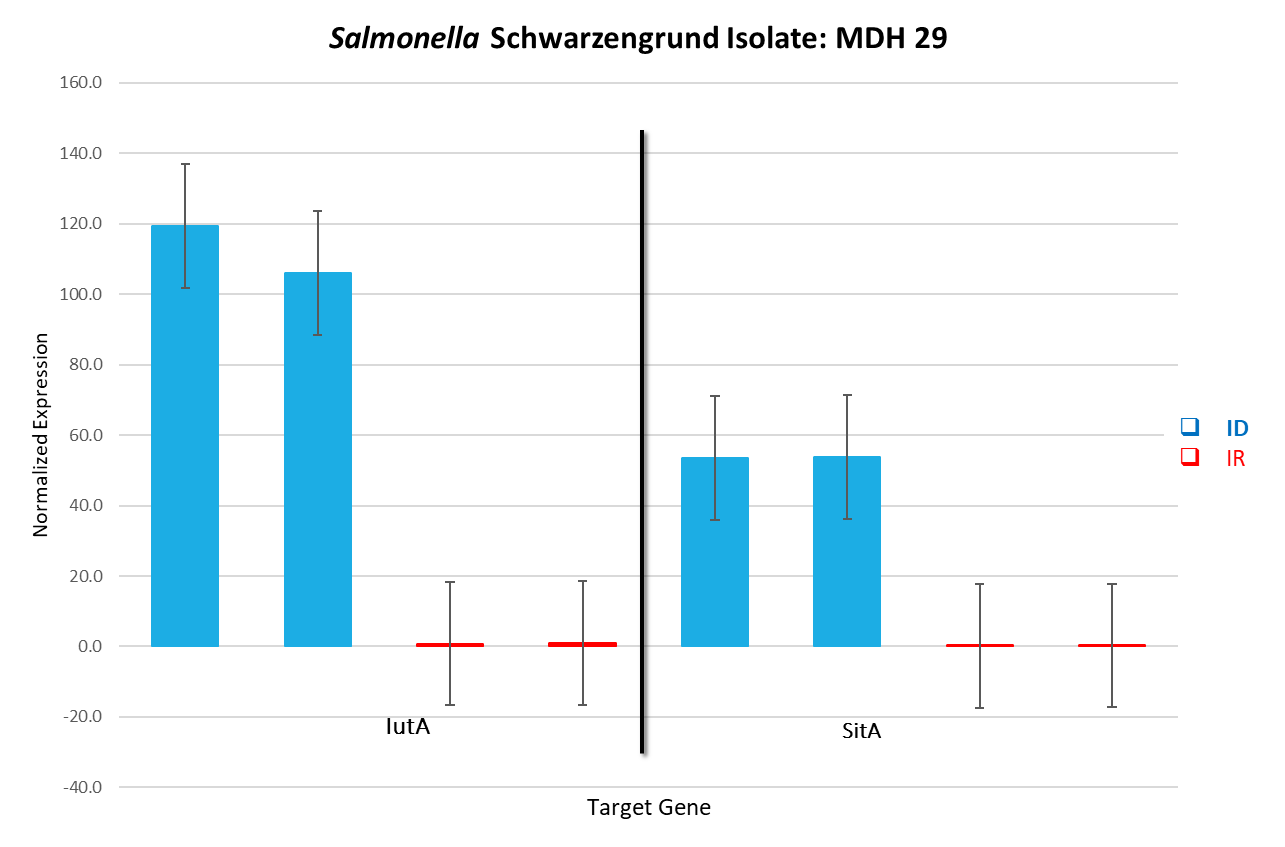


# Figure S2B


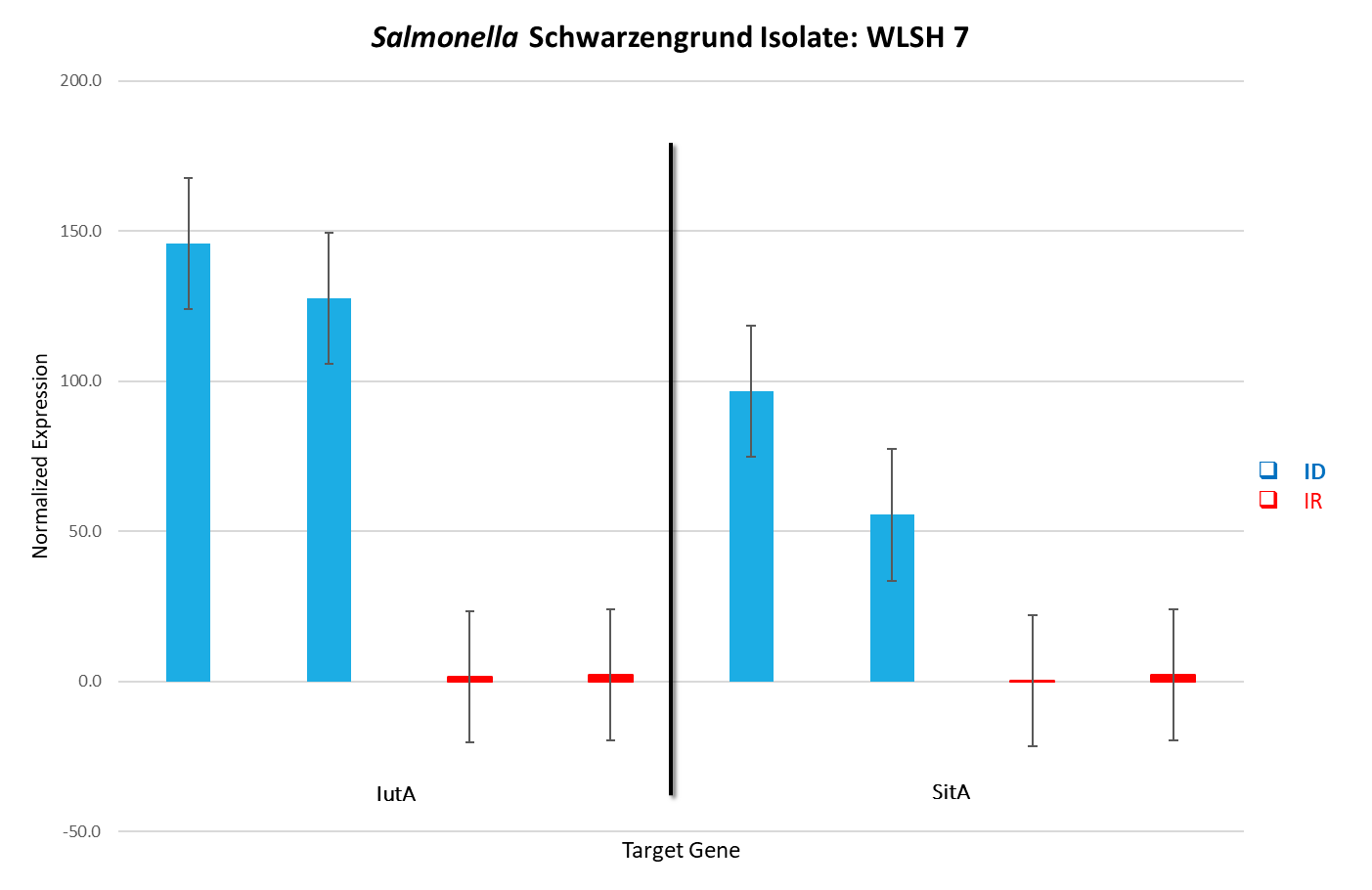


**Figure S2.** Iron acquisition genes of IncFIB carrying *Salmonella Schwarzengrund* isolated from human were upregulated in Iron-depleted conditions compared to Iron-rich. A) *sitA* and *iutA* expression in LBID (blue bar) and LBIR (red bar) for MDH29. B) *sitA* and *iutA* expression in LBID (blue bar) and LBIR (red bar) for WLSH7. Two biological replicates and three technical replicates were used for each sample (+/- SD). Gene expression were normalized with base line expression of control isolate in each gene that were grown in LB. LBID=iron depleted LB; LBIR =iron-rich LB.

**Figure S3**

**Figure S4:**

**Figure S5:**

**Figure S6**
